# Phosphate, Fractures, and Frustration—A Missed Diagnosis of Oncogenic Osteomalacia Leading to Multisystem Complication

**DOI:** 10.1002/ccr3.70790

**Published:** 2025-08-11

**Authors:** Ryan Michael Wilson, Lydia Sturridge

**Affiliations:** ^1^ Emergency Medicine Frimley Park Hospital NHS Foundation Trust Frimley UK

**Keywords:** diagnostic delay, dilated cardiomyopathy, fibroblast growth factor 23 (FGF23), hypophosphatemia, phosphate‐wasting disorder, tumor‐induced osteomalacia (TIO)

## Abstract

Persistent hypophosphatemia must prompt thorough evaluation. This case highlights the severe, multisystem consequences of delayed recognition of oncogenic osteomalacia. Early biochemical assessment, imaging, and multidisciplinary involvement are critical to avoid misdiagnosis and prevent irreversible complications such as skeletal fragility and cardiomyopathy.

## 1 Introduction

Oncogenic osteomalacia, or tumor‐induced osteomalacia (TIO), is a rare paraneoplastic syndrome caused by renal phosphate wasting due to excess fibroblast growth factor 23 (FGF23) which is secreted by phosphaturic mesenchymal tumors (PMTs). This results in hypophosphatemia, osteomalacia, muscular weakness, and bone pain [1]. This condition is often misdiagnosed due to nonspecific symptoms and the difficulty in locating the tumor [1].

This report details the prolonged diagnostic journey of a 44‐year‐old mechanic whose symptoms were misattributed to mechanical causes. It underscores the importance of an early biochemical assessment, imaging, and multidisciplinary collaboration to prevent late‐stage complications.

### 1.1 Prevalence Data for Oncogenic Osteomalacia

Oncogenic osteomalacia is an extremely rare paraneoplastic syndrome, with an estimated global incidence of approximately 0.18 cases per million persons per year, based on epidemiological analysis of case reports and small series worldwide [2]. In the United Kingdom, precise prevalence data are lacking due to under‐recognition and diagnostic delays; however, extrapolation from available international data suggests a similarly low frequency, potentially fewer than 20 active cases diagnosed annually across the UK [3]. The rarity is compounded by the small size and often indolent nature of the PMTs responsible, which frequently evade detection for years. Global registries and long‐term cohort studies remain limited, contributing to the underestimation of true prevalence.

## 2 Case History and Examination

The patient presented with progressive musculoskeletal symptoms—chronic pain, worsening lower‐limb weakness, and height loss—that were repeatedly attributed to prior orthopedic trauma. That trauma stemmed from a motor‐bike accident in late February to mid‐March 2015, followed by removal of ankle metalwork on December 6, 2017. His analgesic regimen consisted of oral Morphine, Amitriptyline, Naproxen, and Gabapentin.

By February 2019 clinic notes had documented “post‐traumatic right‐ankle osteoarthritis,” and by March 2020 the patient's back, hip and ankle pain required permanent use of crutches. During a chest infection admission (July 2021) routine bloods incidentally revealed severe hypophosphatemia of 0.29 mmol/L (reference range: 0.80–1.50 mmol/L), yet no metabolic work‐up was initiated. A second admission during August of 2021 again showed low phosphate at 0.38 mmol/L (reference range: 0.80–1.50 mmol/L); endocrinology commenced Alfacalcidol 250 ng daily on August 5, 2021.

In the months leading up to diagnosis, he developed exertional dyspnoea, paroxysmal nocturnal dyspnoea, and bilateral edema. Cardiovascular work‐up in November of 2021 showed a blood pressure of 155/105 mmHg, BNP > 2000 ng/L (reference range < 125 ng/L), and an electrocardiogram (ECG) demonstrated evidence of left ventricular hypertrophy (LVH) with T‐wave inversion. Transthoracic echocardiography revealed a dilated left ventricle (6.3 cm) with an ejection fraction of approximately 30% (reference range 55%–70%), consistent with dilated cardiomyopathy (DCM).

Musculoskeletal symptoms persisted despite serial phosphate measurements of 0.57 mmol/L (September 2021), 0.67 mmol/L (October 2021), and 0.75 mmol/L (November 2021), alongside rising alkaline phosphatase to > 760 U/L (reference range 30–130 U/L) and a DEXA *T*‐score of −4 (reference range for normal bone density > −1.0) during December 2021. Nevertheless, no metabolic bone assessment was made until further deterioration prompted targeted investigation in early 2022. At this time, total height loss was quantified at 15 cm, from a height of 185 cm to a height of 170 cm. His gait was formally assessed by orthopedics, and despite using two crutches, was described as an antalgic gait with slow, cautious steps and a wide base for stability, and he was unable to ascend to sit on the assessment bed during the consultation. For clarity, given the complex background of this patient, Table 1 illustrates the timeline of events relating to this patient's presentation.

**TABLE 1:** Summarizing the key clinical events in chronological order.

Date	Clinical event
27/02/2015–17/03/2015	Admission for motorbike accident with multiple fractures (left proximal humerus, right distal radius, right ankle)
06/12/2017	Removal of metalwork from right ankle
28/02/2019	T&O letter: chronic right ankle osteoarthritis and back pain (under pain team > 1 year)
13/03/2020	Surgical referral for worsening back and right ankle pain; patient using crutches
16–22/07/2021	Admission for chest infection; phosphate 0.29 mmol/L incidentally identified; PPI stopped
04–05/08/2021	GP surgery admission for low phosphate; endocrinology follow‐up arranged
05/08/2021	Phosphate 0.38 mmol/L; one‐alfacalcidol 250 ng daily started; no full metabolic work‐up initiated despite normal vitamin D and calcium
17/09/2021	GP attributed diarrhea to phosphate tablets (9/day); phosphate malabsorption still attributed to PPI use
15/10/2021	Endocrinology: phosphate 0.67 mmol/L; maintained on 9 phosphate tablets/day
03/11/2021	Endocrinology: phosphate 0.75; phosphate reduced from 9 to 6 tablets/day; DEXA scan arranged
18/11/2021	Initial cardiology review for DCM; suspected phosphate wasting disorder
23/11/2021	Phosphate 0.77 mmol/L; Alfacalcidol increased to 0.75 μg daily; colecalciferol 5000 IU OD started; phosphate dose reduced for tolerance
25/11/2021	Orthopedic referral: unable to straight leg raise (SLR), worsening pain in leg and groin
16/12/2021	Cardiology: nuclear scan and cardiac clearance for surgery
24/12/2021	Phosphate 0.63 mmol/L; ALP reduced to 764 U/L; colecalciferol increased to 10,000 IU OD; phosphate increased to 6 tablets/day
29/12/2021	DEXA scan: *T*‐score –4.0 confirming severe osteoporosis
06/01/2022	PET scan: FGF23‐secreting tumor confirmed (24 x 23 x 21 mm, right gluteus)
11/01/2022	Orthopedics requested MDT advice on biopsy vs. simultaneous hip replacement
14/01/2022	MDT: wide excision agreed
20/01/2022	Clinic letter to GP: histology confirmation required prehip replacement
24/01/2022	Histology: tumor benign; plan for bilateral hip replacements
16/03/2022	Bilateral hip replacements performed
14/03/2022–22/03/2022	Phosphate and calcium replacement paused during surgery
28/03/2022	Endocrinology: phosphate and Alfacalcidol stopped; Vit D and calcium continued
30/05/2022	Endocrinology: phosphate 1.79 mmol/L, PTH 8.7 pmol/L; patient mobile with stick; colecalciferol stepped down after DEXA showed recovery

**TABLE 2:** Demonstrating the key biochemical findings in august 2021.

Test	Result	Reference range
Alkaline phosphatase	504 U/L	30–130 U/L
Phosphate	0.57 mmol/L	0.80–1.50 mmol/L
Parathyroid Hormone (PTH)	5.8 pmol/L	1.6–6.9 pmol/L
Serum Calcium	2.18 mmol/L	2.20–2.60 mmol/L
25‐Hydroxyvitamin D	65 nmol/L	76–250 nmol/L
24‐H urinary phosphate	50 mmol/24 h	15–50 mmol/24 h

### 2.1 Differential Diagnoses, Investigation, and Treatment

**FIGURE 1:**
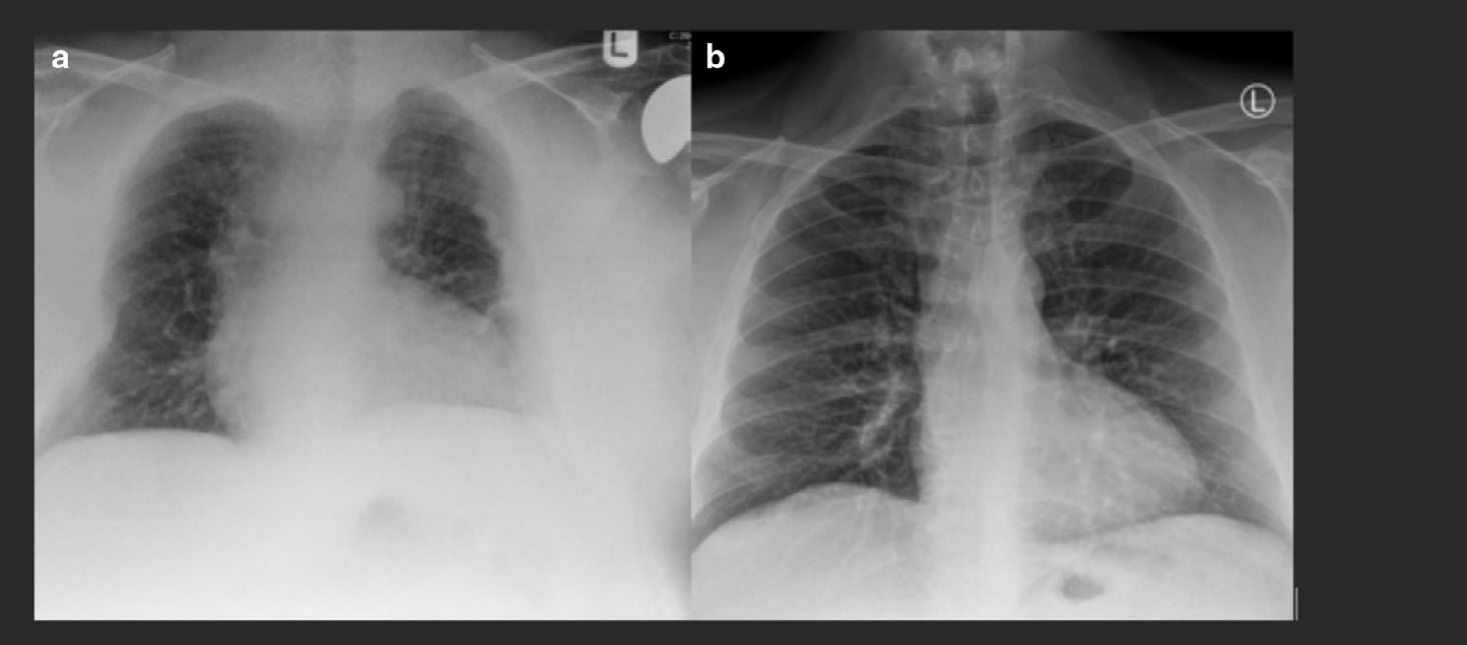
(a, b) Chest radiographs of the patient at time of presentation (left) and 2 years prior (right), demonstrating features detailed below.

The chest radiographs (Figure 1a,b), in themselves, could retrospectively be used to obtain a diagnosis of osteomalacia. It is evident in the left radiograph—when there was established disease—two features that point to this:

- Loss of volume in comparison to the predisease study on the right [4].
- Plaques throughout all three zones in the left lateral lung [5].

His first biochemical abnormality was detected during an emergency‐department attendance for chest tightness and dyspnoea in July 2021: serum phosphate 0.29 mmol/L (reference 0.8–1.5 mmol/L). The result was attributed to long‐term proton pump inhibitor use, and the drug was terminated. A second admission in August 2021 again demonstrated hypophosphatemia (0.38 mmol/L); both Alfacalcidol 250 ng daily was subsequently commenced, and a 24 h urinary phosphate was found to be elevated at 51 mmol/24 h (reference range 15–50 mmol/24 h), yet no comprehensive metabolic work‐up followed.

Weeks after terminating his use of the PPI, his phosphate level did not increase; however, no further investigation was performed, and the patient was discharged with phosphate supplementation.

A full bone profile later that month, which is denoted below in Table 2, yielded the following findings.

At this time, Cholecalciferol supplementation at 5000 IU one daily was introduced alongside his Alfacalcidol, which itself was uptitrated to 0.75 μg daily; however, no further workup for the etiology of the biochemical findings was undertaken.

As pain intensified, imaging began to reveal a metabolic pattern. A contrast pelvic radiograph in early December 2021 showed degenerative change but no clear diagnosis; however, MRI of the spine and pelvis on December 14, 2021 demonstrated bilateral subcapital femoral‐neck fractures, sacral insufficiency fractures, diffuse marrow abnormalities, bilateral acetabular labral tears, and vertebral fractures from T10 to L2. These findings were still misattributed to mechanical stress, and persistently high ALP—peaking at 1025 U/L in late December 2021—was not linked to the ongoing skeletal deterioration. His cholecalciferol was increased to 10,000 IU once daily in light of ongoing manifestations of mineral bone disease to optimize repletion.

After further deterioration and the development of severe skeletal fragility was the possibility of oncogenic osteomalacia (TIO) considered. A FGF23 level was undertaken in early January 2022; this was elevated at 474 RU/mL (reference range < 100 RU/mL). Regretfully, a tubular maximal reabsorption of phosphate in relation to glomerular filtration rate (TmP/GFR) could not be retrospectively obtained.

**FIGURE 2:**
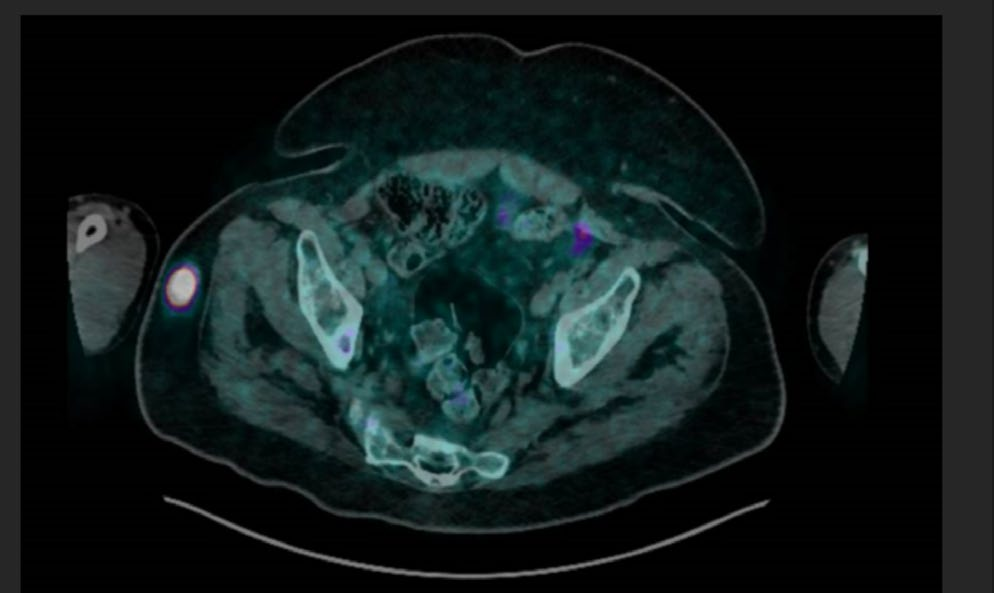
Ga‐68 DOTATATE PET scan localizing the tumor to the right gluteal region.

At this time, the findings were attributed to a phosphate‐wasting disorder, likely due to an FGF23‐secreting tumor. To localize the suspected tumor, an urgent Ga‐68 DOTATATE PET scan was performed (Figure 2) early in the month, demonstrating a highly avid lesion in the right gluteal region (SUV max 30.8, 24 x 23 x 21 mm). While consistent with a PMT, definitive diagnosis was confirmed through histopathological examination postresection [2].

**FIGURE 3:**
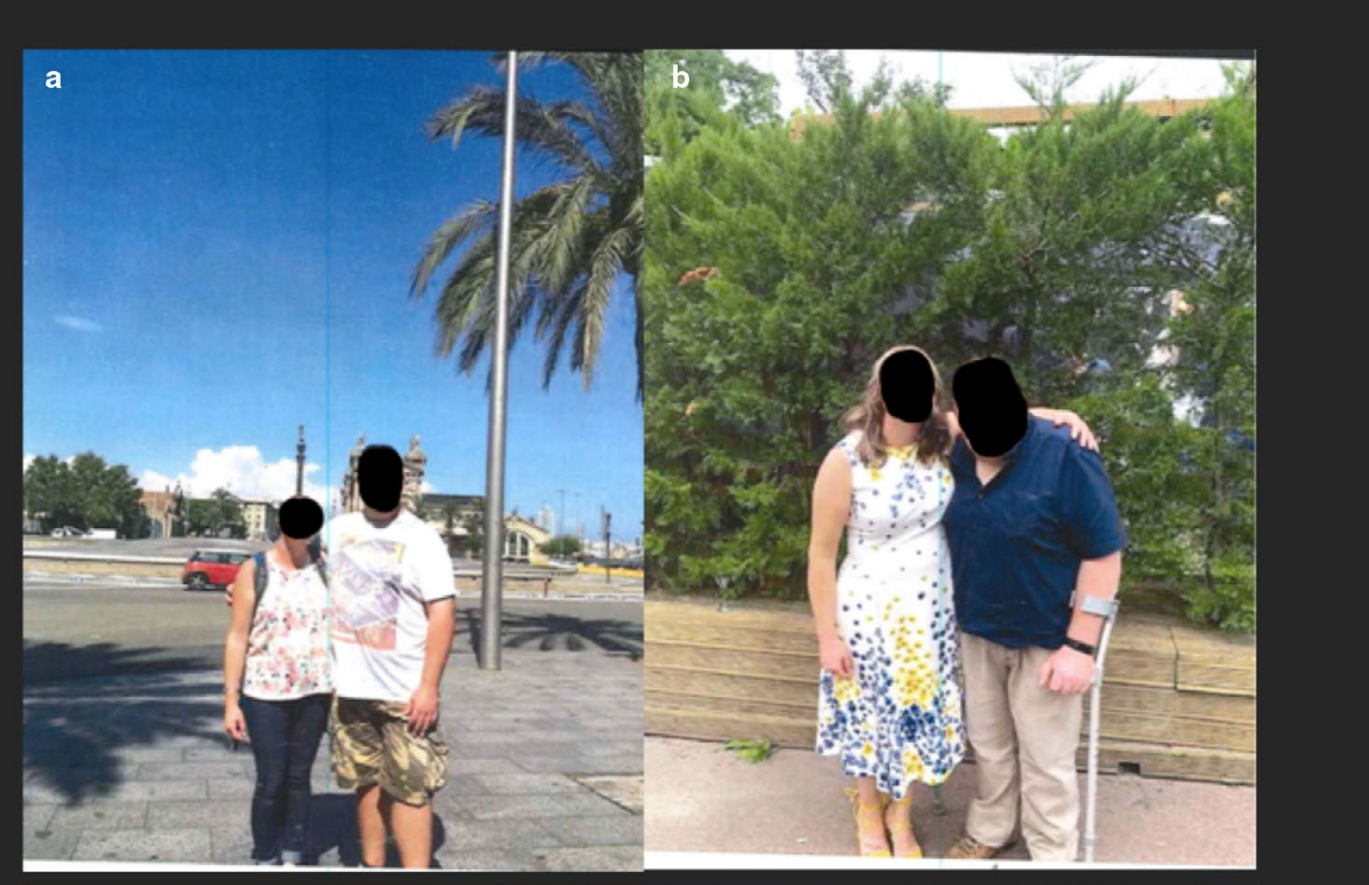
(a, b) Two photographs of the patient, one before the onset of symptoms, and the other after the surgical resection of the tumor and implementation of bilateral total hip replacements. Faces are obscured for confidentiality; the patient is on the right in both photographs. The woman on the left is the same person in both photos.

### 2.2 The Role of FGF23

FGF23 is a key endocrine regulator of phosphate homeostasis, primarily produced by osteocytes and osteoblasts in response to rising serum phosphate and active vitamin D levels. It acts on the kidneys to promote urinary phosphate excretion by downregulating the sodium phosphate cotransporters (NaPi‐IIa and NaPi‐IIc) in the proximal renal tubules, thereby reducing phosphate reabsorption [6]. Simultaneously, FGF23 suppresses renal 1α‐hydroxylase activity, leading to reduced synthesis of 1,25‐dihydroxyvitamin D (calcitriol), which in turn lowers intestinal phosphate absorption [6]. This dual action results in a net hypophosphatemic effect. In oncogenic osteomalacia, PMTs secrete excessive FGF23, producing pathologically high phosphate loss, impaired bone mineralization, and osteomalacia [7].

## 3 Conclusion and Results

With the tumor localized on January 2022 by Ga‐68 DOTATATE PET‐CT, wide excision was performed 3 weeks later, providing curative treatment for oncogenic osteomalacia. Definitive skeletal stabilization followed when bilateral total hip replacements were undertaken during mid‐March 2022. In the interim, a number of measures were implemented:

– August 2021–October 2021: high‐dose phosphate supplementation, titrated up to the equivalent of Sando‐Phos 1 g three times daily (but was later adjusted for tolerance).
– August 2021: initiation of calcitriol analogue (one‐alfacalcidol 250 ng daily), increased to 0.75 μg daily in November 2021 to counteract excess FGF‐23.
– Planned bisphosphonate therapy was scheduled for postsurgical evaluation but was not commenced once biochemical remission was achieved; the rationale for this was not available when retrospectively examining the patient notes.

Although phosphate and active vitamin‐D supplementation were maintained immediately after tumor excision in January 2022, the need for replacement therapy rapidly diminished once renal phosphate handling normalized. During the inpatient period for bilateral hip replacements (Mid‐March 2022) both phosphate tablets and Alfacalcidol were discontinued without biochemical relapse. Endocrinology review later that month formalized this decision, leaving only maintenance calcium and vitamin‐D support. Subsequent follow‐up showed serum phosphate within the reference range (1.79 mmol/L in late May 2022), allowing high‐dose Cholecalciferol (10,000 IU daily) to be stepped down in June 2023 to a physiological maintenance preparation (Adcal D3, one tablet twice daily) after DEXA confirmed skeletal recovery (*Z*‐score improving from −4 to +1).

Following tumor resection and orthopedic optimization, the patient's functional recovery was striking. By late March 2022—within weeks of surgery—he could ambulate 5 miles with a single walking stick, and by 30 May 2022 had discontinued long‐term opioids entirely. Quality‐of‐life scores improved for the first time in years, and his heart failure was reclassified as high‐output; cardiac symptoms diminished in parallel with normalization of phosphate metabolism. The majority of the clinical improvement was elicited with resection of the offending neoplasm, as opposed to with pharmacological therapy.

The postresection echocardiogram performed on 4 June, 2023 demonstrated a normal left ventricular size and an ejection fraction of 60%–65%, with a dilated right ventricle showing preserved function. The left atrium was dilated, but no significant valvular disease or pericardial effusion was noted. Diastolic function was normal, with an E to A ratio of 1.0 and a mitral valve E‐wave peak velocity of 0.58 m per second. Tricuspid annular plane systolic excursion was 2.7 cm, suggesting preserved right ventricular function. No mitral or tricuspid regurgitation was seen, and the aortic valve was tricuspid in morphology with no regurgitation. The thoracic aorta was not visualized. The patient was in sinus rhythm during the scan.

## 4 Discussion

### 4.1 Literature Review

TIO, also known as oncogenic osteomalacia, is a rare paraneoplastic disorder caused by typically benign PMTs that secrete excess FGF23, resulting in renal phosphate wasting, suppressed 1α‐hydroxylase activity, and impaired bone mineralization [1]. Patients commonly present with vague symptoms such as bone pain, proximal muscle weakness, and recurrent insufficiency fractures, which are often misattributed to mechanical or rheumatological causes [8]. These tumors are usually small, slow‐growing, and difficult to detect on routine imaging, leading to significant diagnostic delays. Retrospective studies report median diagnostic delays of 2–4 years, with some cases exceeding a decade. The biochemical hallmark of TIO includes persistent hypophosphatemia, inappropriately low or normal 1,25‐dihydroxyvitamin D, elevated alkaline phosphatase, and raised or inappropriately normal FGF23 levels [1]. Functional imaging—most commonly Ga‐68 DOTATATE PET/CT—is typically required to localize the tumor, with surgical excision offering rapid biochemical resolution and reversal of skeletal pathology.

The critical learning points from this case are detailed below.

### 4.2 Persistent Hypophosphatemia is Never Benign

This gentleman had chronic hypophosphatemia, which was dismissed for months, and persisted after the termination of PPI use; cases of chronic hypophosphatemia should warrant an early biochemical workup, including an early FGF23 measurement in unexplained cases [9].

Delayed diagnosis of oncogenic osteomalacia can lead to prolonged phosphate wasting and impaired bone mineralization, resulting in progressive skeletal deformities, fractures, and chronic pain; while chronic hypophosphatemia has also been associated with adverse cardiovascular outcomes such as LVH and impaired myocardial energetics [10].

### 4.3 Unexplained Fractures Require Metabolic Bone Assessment

This gentleman regretfully suffered a number of fractures and the debilitation associated with this due to a delayed metabolic bone disease diagnosis [11]. His height loss of 15 cm, as evidenced in Figure 3a,b, was reflective of this. Early phosphate testing is essential in the evaluation of unexplained musculoskeletal symptoms, particularly when accompanied by features such as proximal myopathy, fragility fractures, or chronic bone pain. Failure to assess phosphate levels risks overlooking conditions such as TIO, where early biochemical detection could significantly alter the diagnostic trajectory and prevent irreversible skeletal morbidity [6].

### 4.4 Multidisciplinary Team Assessment is Vital

Early involvement of endocrinology, rheumatology, pain medicine, and cardiology is essential in complex cases, particularly when multisystem features such as electrolyte abnormalities, musculoskeletal pain, functional decline, or cardiac dysfunction coexist. Prompt multidisciplinary collaboration improves diagnostic accuracy, expedites localization of the underlying pathology, and optimizes therapeutic planning in conditions such as TIO [11].

### 4.5 Difficulty in Obtaining the Underlying Diagnosis With Pre‐Existing Diagnoses of Chronic Pain Syndrome and Osteoarthritis, in the Context of Biases

The initial working diagnoses of chronic pain syndrome and post‐traumatic osteoarthritis, though superficially plausible given the patient's orthopedic history, ultimately served as red herrings that delayed appropriate metabolic evaluation. His longstanding musculoskeletal pain, reliance on crutches, and previous joint trauma were repeatedly interpreted through a mechanical lens, reinforcing a narrative of degenerative joint disease. However, this perspective failed to reconcile the biochemical abnormalities that emerged over time. Persistent hypophosphatemia—recorded as low as 0.29 mmol/L—alongside rising alkaline phosphatase and vitamin D insufficiency, pointed towards a systemic phosphate‐wasting disorder rather than a localized musculoskeletal process. The lack of response to analgesia, progressive skeletal fragility, and profound osteoporosis (*T*‐score –4.0) further challenged the initial assumptions. These biochemical derangements, particularly in the context of diffuse bone pain and multiple insufficiency fractures, should have prompted earlier consideration of oncogenic osteomalacia, underscoring the importance of integrating metabolic data with clinical history to avoid diagnostic anchoring.

The delayed diagnosis in this case was shaped by both cognitive and systemic biases. Anchoring bias led clinicians to fixate on mechanical causes—such as osteoarthritis and chronic pain—despite worsening symptoms and abnormal biochemistry. This was reinforced by diagnostic momentum, with each new clinician perpetuating existing assumptions without critically reassessing the case [12]. The chronic pain label, in particular, risked minimizing legitimate pathology, while the rarity of oncogenic osteomalacia made it less cognitively available as a differential (availability bias) [13]. Fragmented care across multiple specialties and a lack of multidisciplinary coordination meant that key metabolic clues—persistently low phosphate, elevated alkaline phosphatase, and progressive bone loss—were not integrated. Together, these biases obscured a unifying diagnosis and highlighted how systemic and cognitive pitfalls can delay recognition of rare but serious metabolic bone disease.

### 4.6 Hypophosphatemia and Cardiomyopathy

Emerging evidence has linked chronic hypophosphatemia with the development of dilated cardiomyopathy, likely mediated through impaired myocardial energetics, reduced intracellular adenosine tri‐phosphate (ATP) availability, and disordered calcium handling—all of which compromise myocardial contractility and cellular function [14].

### 4.7 Algorithms for Obtaining a Diagnosis of TIO

A structured diagnostic algorithm for TIO, incorporating serum phosphate, FGF23 testing, TmP/GFR calculation, and Ga‐68 DOTATATE PET/CT imaging, has been recently outlined in international consensus guidance [15].

### 4.8 Clinical Red‐Flags Indicative of Metabolic Bone Disease

Clinical red flags suggestive of underlying metabolic bone disease include: unexplained fragility fractures, diffuse or persistent bone pain, proximal muscle weakness, low‐trauma height loss, delayed fracture healing, and biochemical abnormalities such as hypophosphatemia or raised alkaline phosphatase in the absence of liver dysfunction [16].

### 4.9 Significance of Early Disease Identification

Early diagnosis and timely surgical resection of the offending tumor are critical to prognosis, as they often result in rapid biochemical normalization, reversal of bone demineralization, and restoration of physical function. Delayed intervention, by contrast, increases the risk of irreversible skeletal damage, prolonged disability, and multisystem complications [6].

The potential differentials for hypophosphatemia are broad, and a targeted biochemical workup and thorough history are required to elicit an accurate diagnosis; to this end Table 3 has been included to demonstrate the variety of biochemical findings in relationship to hypophosphatemia.

**TABLE 3:** Demonstrating common differential diagnoses for hypophosphatemia with mechanistic and clinical correlates.

Diagnosis	Mechanism	Key features
Vitamin D deficiency	↓ Intestinal phosphate absorption	Low phosphate and calcium, raised PTH, osteomalacia/rickets
Fanconi syndrome	Proximal tubule defect → phosphate wasting	Glycosuria, aminoaciduria, acidosis, hypokalemia
Chronic alcohol use	Poor intake, absorption, ↑ renal losses	Often coexists with hypomagnesemia, liver dysfunction
Tumor‐induced osteomalacia (TIO)	↑ FGF‐23 → ↓ renal phosphate reabsorption	Elevated FGF‐23, occult mesenchymal tumor, fractures, muscle weakness
Primary hyperparathyroidism	↑ PTH → ↓ phosphate reabsorption	Hypercalcaemia, raised ALP, nephrolithiasis
Refeeding syndrome	Sudden cellular uptake after nutrition restart	Hypophosphatemia, hypokalemia, hypomagnesemia poststarvation
Diabetic ketoacidosis recovery	Cellular shift during insulin therapy	May cause transient profound hypophosphatemia
Sepsis/critical illness	Redistribution and increased cellular demand	Common in ICU; treat if severe
Diuretic therapy (e.g., acetazolamide, thiazides)	↑ Renal losses	Medication history important; self‐limiting on cessation
Malabsorption syndromes (e.g., coeliac disease)	↓ Phosphate absorption due to impaired gut mucosa	May present with concurrent iron, calcium, and vitamin D deficiency
Chronic kidney disease (advanced stages)	↓ Renal phosphate excretion regulation	May see hypophosphatemia in early stages, hypophosphatemia in dialysis or late‐stage due to poor intake, medication effect

*Note:* This table summarizes common causes of hypophosphatemia, their underlying mechanisms, and distinguishing clinical features. It is intended to aid diagnostic evaluation by integrating pathophysiological insight with practical bedside recognition. Mechanisms and features summarized from references [8] [1] [17] [18] [19] [20].

## Author Contributions

**Ryan Michael Wilson:** project administration, writing – original draft, writing – review and editing. **Lydia Sturridge:** writing – review and editing.

## Ethics Statement

Ethical approval was not required for this case report, as per local institutional guidelines, because it describes a single patient with informed written consent obtained for publication. No experimental interventions were conducted outside the scope of routine clinical care.

## Consent

Written informed consent was obtained from the patient for publication of this case report in accordance with the Journal's Patient Consent Policy.

## Conflicts of Interest

The authors declare no conflicts of interest.

## Data Availability

Data available on request due to privacy/ethical restrictions The data that support the findings of this study are available on request from the corresponding author. The data are not publicly available due to privacy or ethical restrictions.
